# Acute respiratory failure in patients with hematological malignancies: outcomes according to initial ventilation strategy. A groupe de recherche respiratoire en réanimation onco-hématologique (Grrr-OH) study

**DOI:** 10.1186/s13613-015-0070-z

**Published:** 2015-09-30

**Authors:** Virginie Lemiale, Matthieu Resche-Rigon, Djamel Mokart, Frederic Pène, Antoine Rabbat, Achille Kouatchet, François Vincent, Fabrice Bruneel, Martine Nyunga, Christine Lebert, Pierre Perez, Anne-Pascale Meert, Dominique Benoit, Sylvie Chevret, Elie Azoulay

**Affiliations:** AP-HP, Hôpital Saint-Louis, Medical ICU, 1 avenue Claude Vellefaux, 75010 Paris, France; Biostatistics Department, Saint Louis Teaching Hospital, Paris, France; ICU, Paoli Calmettes Institut, Marseilles, France; ICU, Cochin Teaching Hospital, Paris, France; Respiratory Unit, Cochin Teaching Hospital, Paris, France; ICU, Angers Teaching Hospital, Angers, France; ICU, Avicenne Teaching Hospital, Bobigny, France; ICU, Mignot Hospital, Versailles, France; ICU, Roubaix Hospital, Roubaix, France; ICU, CH vendée, La Roche sur Yon, France; ICU, Brabois Teaching Hospital, Nancy, France; ICU, Bordet Institut, Brussels, Belgium; ICU, Ghent Teaching Hospital, Ghent, Belgium

**Keywords:** Noninvasive ventilation, Immunosuppression, Leukemia, Lymphoma, Neutropenia, Mechanical ventilation

## Abstract

**Background:**

In patients with hematological malignancies and acute respiratory failure (ARF), noninvasive ventilation was associated with a decreased mortality in older studies. However, mortality of intubated patients decreased in the last years. In this study, we assess outcomes in those patients according to the initial ventilation strategy.

**Methods:**

We performed a post hoc analysis of a prospective multicentre study of critically ill hematology patients, in 17 intensive care units in France and Belgium. Patients with hematological malignancies admitted for ARF in 2010 and 2011 and who were not intubated at admission were included in the study. A propensity score-based approach was used to assess the impact of NIV compared to oxygen only on hospital mortality.

**Results:**

Among 1011 patients admitted to ICU during the study period, 380 met inclusion criteria. Underlying diseases included lymphoid (*n* = 162, 42.6 %) or myeloid (*n* = 141, 37.1 %) diseases. ARF etiologies were pulmonary infections (*n* = 161, 43 %), malignant infiltration (*n* = 65, 17 %) or cardiac pulmonary edema (*n* = 40, 10 %). Mechanical ventilation was ultimately needed in 94 (24.7 %) patients, within 3 [2–5] days of ICU admission. Hospital mortality was 32 % (123 deaths). At ICU admission, 142 patients received first-line noninvasive ventilation (NIV), whereas 238 received oxygen only. Fifty-five patients in each group (NIV or oxygen only) were matched according the propensity score. NIV was not associated with decreased hospital mortality [OR 1.5 (0.62–3.65)].

**Conclusions:**

In hematology patients with acute respiratory failure, initial treatment with NIV did not improve survival compared to oxygen only.

Clinical trial.gov number NCT 01172132

## Background

Acute respiratory failure (ARF) remains the first reason for admission to ICU in patient with hematological disease [1–3]. Various etiologies lead to ARF in that setting. Among the determinants of mortality in hematology patients with ARF, mechanical ventilation remains the major determinant of death [1, 4], as well as the type of ARF etiology (e.g., Invasive aspergillosis) [1, 5], poor performance status, allogeneic bone marrow stem cell transplantation, delayed ICU admission [6] or associated organ dysfunction [1, 3, 7]. Fifteen to 20 years ago, hematology patients with acute respiratory failure exhibited mortality rates of about 50 % [7–11], and for those who needed mechanical ventilation mortality reached 90 % [8, 12]. At that time, studies reported significant survival benefits from noninvasive ventilation [9, 12], even though delayed intubation after NIV failure was associated with higher mortality [11, 13]. In that setting, noninvasive ventilation (NIV) was an efficient alternative to invasive mechanical ventilation (iMV). In 2001, a randomized controlled trial of NIV versus oxygen in 52 immunocompromised reported a significantly decreased mortality when NIV was applied [12]. In that study, mortality of cancer patients with acute respiratory failure not receiving NIV was 93 %. Another study in post-operative solid organ transplant patients also reported survival benefits from early NIV [14]. However, more recently, non-randomized studies failed to confirm these results [15]. Over the last two decades, survival of patients with hematological malignancy admitted to ICU improved, even for patients receiving mechanical ventilation [16–19]. For instance, in a recent study from our GRRROH network, mortality of hematology patients requiring mechanical ventilation and who had at least one additional organ dysfunction was 60 % [1]. Similar results were reported by others [20, 21]. Also, survival in cancer patients with ARDS increased from 18 to 48 % over the last 20 years [4]. Therefore, survival benefits from NIV could either be harder to demonstrate or may have been balanced by improvements in the way mechanical ventilation is delivered [22, 23]. Hence, to appraise the literature with more recent prospective multicenter data, we assessed the impact of NIV use on mortality in a cohort of hematology patients admitted to the 17 ICUs for acute respiratory failure.

## Methods

### Data source

This study is a post hoc analysis of a prospective cohort of 1011 patients admitted to ICU with hematological malignancy [1]. This cohort was prospectively recruited between 01/2010 and 05/2011 from 17 ICUs in France and Belgium. All patients with hematological malignancies admitted to ICU were included in the cohort and data were prospectively collected every day from admission to day 28. Data reported in tables and figures were collected prospectively by study investigators. Hospital mortality was available for all the patients.

### Selection of the study population

Among the 1011 hematology patients, those admitted with ARF were included in the present analysis. Inclusion criteria were presence of ARF as defined by tachypnea >30/min, respiratory distress, SpO2 <90 at admission and labored breathing. Exclusion criteria were mechanical ventilation at admission.

### Variables of interest

Underlying disease, performance status in the 3 months from ICU admission, malignancy status (remission or not), ARF etiology, severity of organ dysfunction were the variables of interest as the primary study identified those as independently associated with hospital mortality [1].

Using pre-established diagnostic criteria [24], three independent investigators analyzed the charts to classify patients as having pulmonary infection, cardiac pulmonary edema, pulmonary infiltration by the malignancy, or other ARF etiologies. Patients were deemed to have an undetermined ARF etiology when no cause of ARF could be clinically or microbiologically documented [24].

### Statistical analyses

All data are presented as medians (25th–75th percentiles) for quantitative variables and frequencies (percentage) for qualitative variables. Organ dysfunction was assessed by dichotomizing the LOD score at day 1 (LOD = 0 or LOD > 0). Baseline characteristics were compared between survival and dead patients using Wilcoxon rank-sum test for quantitative variables and Fisher’s exact test for qualitative variable.

A propensity score-based approach was used to limit bias of between-group comparison to assess the impact of NIV compared to oxygen only on hospital mortality [25]. The propensity score was defined as the probability that a patient with specific baseline characteristics receives NIV trial. Then, two patients with identical propensity score value but in the two different treatment groups (NIV versus oxygen only) can be considered as comparable, and matching on the propensity score has been shown as one of the most efficient methods for treatment effect assessment [26, 27]. We computed the propensity score using logistic regression to predict NIV O2 group based on baseline characteristics known to be linked to the mortality [2] (underlying hematological disease, performances status over 2, delay between hospital admission and ICU admission, delay since the diagnosis, complete or partial remission, allogenic stem cell transplantation, admission from ICU) or with a standardized difference above 0.1 (age over 60 years, gender, neutropenia, etiology of acute respiratory failure, respiratory SOFA score over 3, kidney SOFA score over 3, hemodynamic SOFA score over 3, SOFA score at day 1 over 7, maximal respiratory rate) [1, 26, 28]. Standardized differences are used to compare balance in baseline covariates between two Oxygen and NIV groups [29]. A 1:1 matching algorithm without replacement was used within a given range of 0.20 standard deviations of the logit of the estimated propensity score [13]. Final analyses on the matched dataset were performed using a logistic regression with a random effect on the paired observations except for the length of stay analyzed with a Cox random effect model. Results were presented as Odds-Ratio (OR) with their 95 % CI. Finally, we performed a sensitivity analysis using the inverse probability weighting (IPW) approach to estimate the treatment effect. This approach consists in using weights based on the propensity score to create a synthetic sample in which the distribution of measured baseline covariates is independent of treatment assignment. All tests were two-sided at the 0.05 significance level. Analyses were performed using R statistical package (online at http://www.R-project.org).

## Results

**Fig. 1:**
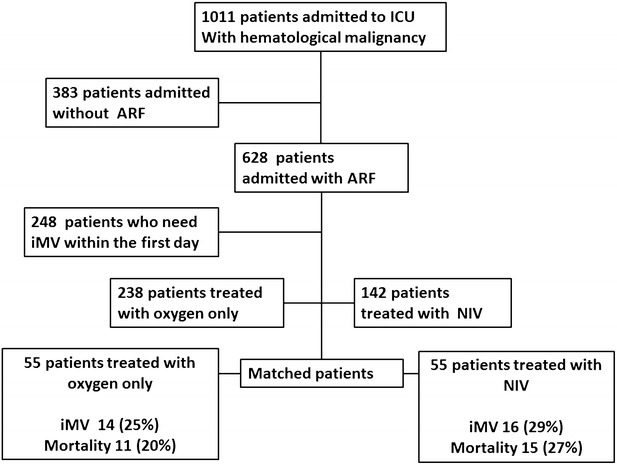
Flow chart. *ICU* intensive care medicine, *ARF* acute respiratory failure, *NIV* noninvasive ventilation, *iMV* invasive mechanical ventilation

Among 1011 patients included in the primary study, 380 were admitted for respiratory symptom and were not requiring mechanical ventilation at ICU admission (Fig. 1). As shown in Table 1, performance status was 0–1 for 308 patients (81 %), the malignancy was active (ongoing/recent chemotherapy) in 265 patients (72.8 %) and 112 (29.4 %) patients were neutropenic at admission. Also, 74 (19.5 %) patients underwent allogeneic stem cell transplantation. ICU admission occurred 5 (0–20) days after hospital admission and 90 (23.7 %) patients were admitted from the emergency department.

**Table 1:** Characteristics of patients admitted with ARF according to initial ventilator strategy

Variables	Patients receiving oxygen only (*n* = 238)	Patient receiving NIV (*n* = 142)	*p*	Std diff
Baseline characteristics				
Age (year) *m*, [IQR]	60 [49–67]	60 [50–70]	0.15	0.14
Gender male	102 (42)	57 (40)	0.67	0.06
Underlying disease			0.27	
Myeloid disease	84 (35)	57 (40)		0.10
Lymphoid disease	109 (46)	53 (37)		0.17
Other	45 (19)	32 (22)		0.09
Delay from diagnosis to ICU admission				
Newly diagnosed	85 (36)	30 (21)		
Remission	58 (24)	41 (29)	0.05	
No remission	91 (38)	41 (29)		
Allogenic stem cell transplantation	41 (17)	33 (23)	0.18	0.15
Performance status >2 (severely disabled or bedridden)	36 (15)	36 (25)	0.015	0.26
Charlson comorbidity score	4 [2–5]	4 [3–5]	0.73	0.03
Delay from hospital to ICU admission (days)	4 [0–19]	7 [1–21]	0.29	0.13
Admission from emergency department	61 (26)	29 (20)	0.26	0.12
Neutropenia at admission	65 (27)	47 (33)	0.25	0.13
ARF etiology			0.20	
Infection	104 (43)	57 (40)		0.07
Cardiogenic edema	21 (9)	22 (15)		0.20
Malignant Infiltration	39 (16)	26 (18)		0.05
Other^a^	74 (31)	37 (26)		0.11
Maximum respiratory rate at day 1	31 [25–36]	35 [30–41]	<0.001	0.57
Shock at day 1	40 (17)	23 (16)	1	0.02
Acute kidney injury at day 1	40 (17)	23 (16)	1	0.02
SOFA score at day 1 > 7	34 (15)	47 (36)	<0.001	0.51
Do not intubate order day 1 or day 2	7 (2.9)	8 (5.6)	0.23	0.09
VNI parameters				
Number of trial/day (day1)		2.5 (2–4)		
Number of trial/day (day2)		2 (2–5)		
Length of NIV (h) (day 1)		4 (2–8)		
Length of NIV (h) (day 2)		5 (3–9)		
Respiratory rate under NIV at day 1		26 (20–32)		
Pressure support day 1 (cm H_2_O)		10 (8–12.7)		
Pressure support day 2 (cm H_2_O)		10 (8–14)		
PEEP at day 1 (cm H_2_O)		5 (5–7)		
PEEP at day 2 (cm H_2_O)		5 (5–6)		
Outcome				
Intubation throughout the ICU stay	48 (20)	46 (32)	0.01	
Time from admission to intubation	3 [2–5]	3 [2–5]	0.85	
ICU length of stay	5 [2–9]	7 [4–20]	<0.001	
ICU-acquired infection	27 (11)	21 (15)	0.34	
Hospital mortality	61 (26)	62 (44)	<0.001	
Hospital mortality of intubated patients	27/48 (56)	35/46 (76)	0.05	

All data are presented as medians [25th–75th percentiles] for quantitative variables and frequencies (percentage) for qualitative variables. Comparisons between the two groups were performed with Chi-square test for qualitative value and Wilcoxon test for quantitative value

*SOFA score* Sequential Organ Failure Assessment score

^a^Other included undetermined diagnosis (*n* = 94, 84.6 % of other diagnosis)

Underlying diseases were acute myeloid leukemia (*n* = 112, 29.5 %), acute lymphoid leukemia (*n* = 28, 7 %), lymphoma (*n* = 108, 28.4 %), myeloma (*n* = 54, 14.2 %), or chronic malignancies (*n* = 78, 20.5 %). ARF etiologies included infection (*n* = 161, 42.4 %), cardiac pulmonary edema (*n* = 43, 11.3 %), pulmonary infiltration by the malignancy (*n* = 65, 17.1 %). No diagnosis was found for 94 (24.7 %) patients and 17 (4.4 %) patients had other miscellaneous diagnosis.

During the first 2 days, 238 (62.6 %) patients received oxygen only and 142 (37.1 %) patients received NIV. Hospital mortality was 32.4 % (*n* = 123) and was higher in the NIV group (44 versus 26 % in the oxygen group, Table 1). Overall intubation rate was 24.7 % (94 patients). Intubation was needed in 46 (32.4 %) patients from the NIV group and 48 (20.2 %) patients from the oxygen group. Table 1 describes time between ICU admission and intubation, ICU length of stay and ICU-acquired infection rate in the NIV group and in the oxygen group. In the NIV group, 8 (5.6 %) patients received high flow nasal cannula between NIV sessions. In the oxygen group, 7 (2.9 %) received high flow nasal cannula.

Patient’s characteristics are reported in Table 2. NIV parameters are described in Tables 1 and 3.

**Table 2:** Patient’s characteristics according to hospital survival status

Variables	Alive at hospital discharge (*n* = 257)	Died before hospital discharge (*n* = 123)	*p*
Baseline characteristics			
Age (year) *m*, [IQR]	60 [50–68]	60 [49.5–68.5]	0.91
Gender male (%)	108 (42)	51 (41)	1
Underlying malignancy			0.61
Myeloid disease	92 (35.8)	49 (39.8)	
Lymphoid disease	114 (44.4)	48 (39)	
Other	52 (19.8)	26 (21.1)	
Disease status at ICU admission			0.93
Newly diagnosed	83 (32.4)	32 (26.2)	
Remission	68 (26.6)	31 (25.4)	
No remission	104 (40.6)	55 (45.1)	
Allogeneic stem cell transplantation	37 (14.4)	37 (30.3)	0.0005
Performance status >2 (severely disabled or bedridden)	39 (14.8)	34 (27.6)	0.005
Charlson comorbidity score	4 [2–5]	3 [3–5]	0.9
Time (days) from hospital to ICU admission	3 [0–16]	12 [2–24.2]	<0.001
Admission from emergency department	72 (28)	18 (14.6)	0.004
Neutropenia at ICU admission	65 (25.3)	47 (38.2)	0.01
ARF etiology			0.49
Infection	115 (44.7)	46 (37.4)	
Cardiogenic edema	29 (11.3)	14 (11.4)	
Malignant Infiltration	40 (15.6)	25 (20.3)	
Other^a^	73 (28.4)	38 (30.9)	
Maximum respiratory rate at day 1 (/min)	32 [26–37]	33.5 [29–40]	0.026
Noninvasive ventilation at day 1 or 2	80 (31.1)	62 (50.4)	0.0004
Shock at day 1	44 (17.1)	19 (15.4)	0.77
Acute kidney injury at day 1	44 (17.1)	6 [4–8]	0.77
SOFA score at day 1 > 7	44 (18)	37 (33)	0.003
Outcome			
Intubation throughout the ICU stay	32 (12.4)	62 (50.4)	<0.001
Time from admission to intubation	4 [2–5]	3 [2–5]	0.77
ICU length of stay	5 [3–7]	7 [3–15]	<0.001
ICU-acquired infection	17 (7)	31 (25)	<0.001

All data are presented as medians [25th–75th percentiles] for quantitative variables and frequencies (percentage) for qualitative variables. Comparisons between the two groups were performed with Chi-square test for qualitative value and Wilcoxon test for quantitative value

*iMV* invasive mechanical ventilation, *SOFA score* Sequential Organ Failure Assessment score

^a^Other included undetermined diagnosis (*n* = 94, 84 %)

**Table 3:** Characteristics of patients matched based on the propensity score

Variables	Oxygen therapy (*n* = 55)	NIV therapy (*n* = 55)	Std diff
Baseline characteristics			
Age (year) *m*, [IQR]	60 [47–67]	61 [49.5–68]	0.05
Gender male (%)	23 (41.8)	20 (36.6)	0.11
Underlying malignancy			
Myeloid disease	27 (49)	22 (40)	0.18
Lymphoid disease	18 (32.7)	19 (34.5)	0.04
Other	10 (18.1)	14 (25.4)	0.17
Remission	16 (29)	18 (32.7)	0.08
Allogeneic stem cell transplantation	10 (18.8)	10 (18.8)	0
Performance status >2 (severely disabled or bedridden)	12 (21.8)	11 (20)	0.04
Charlson comorbidity score	4 [3–6]	4 [3–5]	0.01
Time (days) from hospital to ICU admission	4 [0–15]	6 [0–13.5]	0.02
Admission from emergency department	39 (71)	40 (72.7)	0.04
Neutropenia at ICU admission	39 (71)	37 (67.3)	0.08
ARF etiology			
Infection	20 (36.4)	20 (36.4)	0
Cardiogenic edema	10 (18.2)	8 (14.5)	0.09
Malignant Infiltration	13 (23.6)	12 (21.8)	0.04
Other^a^	12 (21.8)	15 (27.3)	0.13
Maximum respiratory rate at day 1/min	33 [26.5–38.5]	32 [29–41]	0.14
Shock at day 1	9 (16.4)	9 (16.4)	0
Acute kidney injury at day 1	9 (16.4)	9 (16.4)	0
SOFA score at day 1 > 7	15 (27.3)	15 (27.3)	0
Do not intubate order day 1 or day 2	0 (0)	2 (3.6)	0.27
VNI parameters			
Number of trial/day (day 1)		3 (2–5)	
Number of trial/day (day 2)		3.5 (1–5.2)	
Length of NIV (hours) (day 1)		6.2 (3–9)	
Length of NIV (hours) (day 2)		5.5 (3–9)	
Respiratory rate under NIV at day 1		26.5 (21.5–32.5)	
Pressure support day 1 (cm H_2_O)		10 (8–13)	
Pressure support day 2 (cm H_2_O)		11.5 (8.5–14)	
PEEP at day 1 (cm H_2_O)		6 (5–7)	
PEEP at day 2 (cm H_2_O)		5 (5–7)	
Outcome			
Intubation throughout the ICU stay	14 (25.4)	16 (29.1)	
Time from admission to intubation	4 [2–6]	3 [2–5]	
Length of ICU stay	5 [3–11]	6 [4–14]	
ICU-acquired infection	5 (9)	6 (11)	
Hospital mortality	11 (20)	15 (27.3)	
Mortality of intubated patients	5/14 (36)	7/16 (44)	

All data are presented as medians [25th–75th percentiles] for quantitative variables and frequencies (percentage) for qualitative variables

Matching criteria were based on baseline characteristics known to be linked to the mortality or with a standardized difference above 0.1

^a^Other included undetermined diagnosis

One hundred ten patients (55 patients in each group) were included in the propensity score (Table 3). Impact of NIV was not different in the matched population for hospital mortality (*p* = 0.37) intubation rate (*p* = 0.67), ICU length of stay (*p* = 0.47), ICU-acquired infection rate (*p* = 0.59) (Table 3). Odd ratio of mortality associated with NIV was 1.50 (0.62–3.65) (*p* = 0.37). A sensitivity analysis conducted with inverse probability weighting approach for propensity score analysis which considers the entire group of 380 patients led to similar conclusions [OR 1.05 (0.49–2.26), *p* = 0.89]. Also, we performed the same matching analysis without patient with ARF related to cardiogenic edema and odd ratio was 1.88 (0.71–5.00), *p* = 0.50.

## Discussion

Acute respiratory failure is the leading cause for ICU admission in patients with hematological malignancies. Mortality of patients requiring mechanical ventilation remains high so that every strategy that avoids intubation should be given priority. Previous studies have demonstrated benefit from early NIV in immunocompromised patients with acute respiratory failure [12] or in post-operative respiratory distress in solid organ transplants [14]. However, at that time, intubation and mortality rates of patients treated in the control group were high as this occurred prior to recent advances in outcomes [17–19]. In this study where overall intubation and mortality rates were 24.7 and 32.4 %, respectively, noninvasive ventilation did not reduce hospital mortality and did not reduce intubation rates. These findings are in agreement with recently published data [12, 14, 15]. They also raise concern about the place dedicated to NIV in hematology patients. As this study did not report any harm from NIV, clinicians should apply NIV as they are used to do, until the results of a trial of NIV versus oxygen become available. NIV remains then the gold standard for the initial ventilatory strategy in hematological patient. However, clinicians should be aware that as mortality rates have dramatically decreased over the last two decades, hematology patients with acute hypoxemic respiratory failure should be managed as are managed all other patients.

Interestingly, patients managed in this study had similar severity at ICU admission than those admitted in other studies [1, 21, 30], but their intubation rate was only 24.7 %, compared to the 40 % previously reported. Early admission may explain some of these differences as patients in the present cohort were admitted 5 (0–20) days after hospital admission, earlier than in previous studies. Therefore, it is another indirect association between early admission and improved outcomes [6, 31]. A trial of early ICU admission remains, however, warranted. Along this line, the low mortality rate reported in this study in ARF patient not intubated offers opportunities for further improving outcomes in this high-risk group.

Over the last decade, mortality of patients with hematological malignancy receiving mechanical ventilation has decreased to reach a plateau of 50–60 % [1, 20]. In that context, NIV failure has been associated with higher mortality [7, 30]. Delayed admission to the ICU in hematology patients with ARF was also associated with high mortality in recent studies [6, 32]. In the present study, propensity score analysis in matched population reported that NIV use was not associated with changes in mortality rates or in any secondary endpoint. In this study, the 32 % mortality rate was relatively low. This difference could be explained by the matching approach having selected observations on their propensity score to receive NIV or oxygen therapy only. Therefore, compared to previous studies, patients with associated organ dysfunction or with need for rapid intubation were excluded, even though they were maintained in other studies. However, we also performed an analysis based on inverse probability weighting. In this analysis, all patients were included and the results were not different. Again, we argue that low mortality rates in our study were related to early admission to the ICU as well as to recent improvements in the management of hematology patients in the ICU. ARF etiology has been shown as a main determinant of outcomes [5, 7]. In the present study, ARF etiology was included in the matching criteria. In that setting, patients likely to have cardiac pulmonary edema would have received more NIV and their outcomes would have been better than patients with other ARF etiologies. However, we performed the analysis without patients with ARF related to cardiogenic edema and conclusion was not different. However, herein, most of ARF were related to infections a condition that has not been reported to be improved by NIV. Similarly, based on the Charlson comorbidity score, very few patients had COPD and none of them were hypercapnic, another situation where NIV should not be discussed. Most of patients in this study were intubated within the first days of admission.

This study had several limitations. First, this was an analysis of a cohort and not a randomized trial aimed to demonstrate benefit from NIV in hematology patients with ARF. Although we performed an analysis based on a propensity score, the results of such trial remain warranted. Second, the decision to offer NIV to ARF patients was left to physician in charge. Even though centers participating to this study have large experience of dealing with hematology patients, no NIV protocol was applied in this study. Third, only 110 patients were included in the propensity analysis. Only one-third of the cohort could be included in the propensity score and is related to different population at baseline. NIV sessions would be prescribed for the most severe patients as shown in Table 1. Maximum respiratory rate at day 1 and SOFA score >7 at day 1 were higher in NIV. Although this sample could be seen as small, it allowed a pseudo-randomisation (in a homogeneous sample) including far more patients than in the 15-year-old studies that demonstrated benefits from NIV. In observational studies, propensity analysis with such a matching procedure ensures to be as close as possible to a randomized clinical trial by selecting patient with comparable characteristics. The result of a sensitivity analysis conducted with inverse probability weighting approach for propensity score analysis which considers the entire group of 380 patients gave a quite different result [OR 1.05 (0.49–2.26) versus 1.50 (0.62–3.65)], but led to similar conclusion. A trial to demonstrate survival benefits from NIV would require the inclusion of at least 300 patients (150 in each group) based on mortality rates reported in this study and in the most recent papers of the literature.

## Conclusion

This study demonstrated no benefit from NIV in a cohort of patients with hematological malignancies admitted to the ICU for acute respiratory failure. The propensity analysis as well as the inverse probability weighting approach suggests that few biases explain this lack of benefit. A trial of early NIV in immunocompromised patients with acute respiratory failure is warranted. Until the results of such trial, clinicians should not deprive hematology patients from early intubation and optimal ventilation [33].

## Appendix

The following institutions participated in this study: Medical ICU, Saint-Louis Teaching Hospital, Paris, France; Medical ICU, Paoli Calmette Institute, Marseille, France; Medical ICU, Cochin Teaching Hospital, Paris, France; Medical ICU, Angers Teaching Hospital, Angers, France; Medical ICU, Pitié-Salpêtrière Teaching Hospital, Paris, France; Medical ICU, Avicenne Teaching Hospital, Bobigny, France; Medical ICU, Roubaix Hospital, Roubaix, France; Medical ICU, Mignot Hospital, Versailles, France; Medical ICU, Hôtel-Dieu Teaching Hospital, Paris, France; Medical ICU, La Roche sur Yon Hospital, La Roche sur Yon, France; Medical ICU, Nancy Teaching Hospital, Nancy, France; Medical ICU, Brest Teaching Hospital, Brest, France; Medical ICU, Jules Bordet Institute, Brussels, Belgium; Medical ICU, Ghent University Hospital, Ghent, Belgium; Medical ICU, Grenoble Teaching Hospital; Medical ICU, Lille Teaching Hospital France; Medical ICU, Saint-Etienne Teaching Hospital, Saint-Etienne, France.
